# Translating Pharmacogenomics: Challenges on the Road to the Clinic

**DOI:** 10.1371/journal.pmed.0040209

**Published:** 2007-08-14

**Authors:** Jesse J Swen, Tom W Huizinga, Hans Gelderblom, Elisabeth G. E de Vries, Willem J. J Assendelft, Julia Kirchheiner, Henk-Jan Guchelaar

## Abstract

Pharmacogenomics is one of the first clinical applications of the postgenomic era. It promises personalized medicine rather than the established “one size fits all” approach to drugs and dosages. The expected reduction in trial and error should ultimately lead to more efficient and safer drug therapy. In recent years, commercially available pharmacogenomic tests have been approved by the Food and Drug Administration (FDA), but their application in patient care remains very limited. More generally, the implementation of pharmacogenomics in routine clinical practice presents significant challenges. This article presents specific clinical examples of such challenges and discusses how obstacles to implementation of pharmacogenomic testing can be addressed.

## Abstract 2

Jesse J. Swen and colleagues review the limited successes of pharmacogenomics (individualized drug dosing based on hereditary factors) and discuss the remaining challenges to its widespread clinical use.

## Introduction

In 2003 the International Human Genome Sequencing Consortium declared that the Human Genome Project had been completed, raising expectations of clinical application in the near future. Pharmacogenomics (PGx) (here used synonymously with pharmacogenetics [Box 1]), promising the end of “one size fits all” drugs and of trial and error in pharmacotherapy, is often predicted to be one of the first such applications [1].

**FIVE KEY PAPERS IN THE FIELD**

**Van den Akker-van Marle et al., 2006** [26] Provides evidence that TPMT genotyping prior to thiopurine treatment initation is cost effective in children with acute lymphoblastic leukemia.

**Gardiner et al., 2005** [4] The authors demonstrate that PGx tests for drug metabolizing enzymes are rarely performed in clinical practice.

**Sconce et al., 2005** [41]. In this paper VKORC1 genotype is combined with CYP2C9 genotype to explain outcome of warfarin treatment.

**Phillips et al., 2004** [22] Systematic review of literature data concerning the cost effectiveness of PGx interventions.

**Kirchheiner et al., 2001** [30] First paper on deriving dose recommendations from pharmacokinetic study data.

The concept of interindividual differences in drug response was proposed as early as 1909 by Garrod in his book *The Inborn Errors of Metabolism* [2]. Today, the concept of PGx, namely that variation in drug response is related to genetic variation, is widely recognized. Two commercially available PGx tests that support the personalization of drug treatment have already received FDA approval. The tests detect variations in the genes coding for enzymes involved in drug metabolism: cytochrome P450 CYP2C19 and CYP2D6 (Roche AmpliChip, http://www.roche.com/), and UDP-glucuronosyltransferase (Invader UGT1AI Molecular Assay; Third Wave Technologies, http://www.twt.com/). Examples of these and other PGx tests actually being used in patient care are sparse, however. Recent surveys in Germany and Australia reported that only a small number of laboratories offer PGx testing for clinical use [3,4]. Current and potential future uses of PGx tests are summarized in Table 1.

**Table 1:**
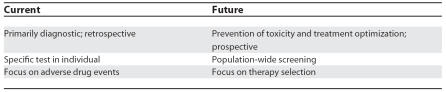
Use of PGx in Clinical Practice

This article focuses on challenges in the translation of PGx to clinical practice. Six challenges associated with consecutive phases in the translation process are discussed (Figure 1). Each of the identified challenges is exemplified by situations from clinical practice, and possible approaches to overcome them are discussed.

**Figure 1:**
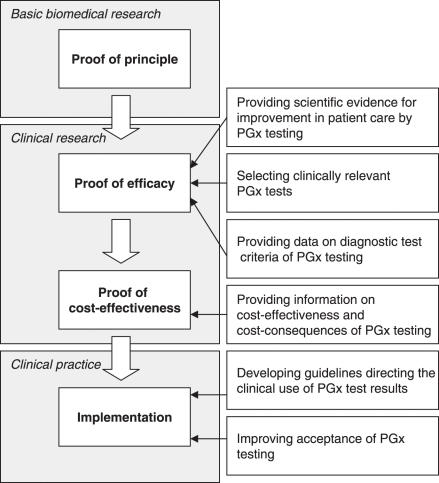
Consecutive Phases and Associated Challenges on the Road to Clinical Implementation of Pharmacogenomics

## Players in the Field

In the challenges presented in Figure 1, several “players” can be identified [5], including the biotechnology and analytical industry, the pharmaceutical industry, research institutions, funding agencies, regulatory agencies, clinicians, and patients. These players each have substantial roles, both individually and in collaboration, in developing and implementing clinical applications of PGx.

As an early step in this process, the biotechnology and analytical industry must develop fast, reliable, and affordable assays for routine PGx measurement. The reaction of the pharmaceutical industry to the concept of PGx has been reserved, possibly because of the potential for market segmentation and an end to the era of blockbuster drugs (Box 2) [5]. Nonetheless, a 2001 report stated that by applying genomics technologies, the investments to develop a drug could be reduced by as much as $300 million and two years [6]. Further, the influence of the pharmaceutical industry on the translation of PGx to the clinic, although considerable, should not be overestimated. Manufacturers can be expected to pursue development of PGx tests only for new compounds and not for drugs already marketed. The latter would most likely be of interest to research institutions, for example academic medical centers.

Indeed, most of our PGx knowledge comes from clinical studies initiated by research institutions. The importance of adequately designed original studies on associations between genetic variation and clinical drug response needs to be recognized by funding agencies, including health insurers and governmental agencies [7]. In recent years, many projects have been funded, and even prospective studies on dose recommendations are now being performed. In addition, these agencies will have to be convinced to reimburse routine PGx testing, which will require extensive information on cost-effectiveness and cost-consequences of PGx testing.

Regulatory agencies, such as the European Agency for the Evaluation of Medicinal Products and the FDA could play a role by recommending or requiring PGx testing for certain drugs, which would obviously provide a strong stimulus. In 2004 and 2005 the FDA approved label changes of 6-mercaptopurine and irinotecan to include PGx information; recommendations for other drugs, such as warfarin, may follow [8,9]. In the case of irinotecan, however, results not fully supporting the dose adjustment included in the label change have been reported [10]. To date, mandatory testing is mentioned only in the package insert of trastuzumab [11]. The FDA has issued a guidance for industry on the subject of PGx and is encouraging voluntary data submission [12]. More recently the FDA and the European Agency for the Evaluation of Medicinal Products have issued a joint procedure for the voluntary submission of PGx data [13].

Following the increase of evidence of clinical relevance and number of available tests, physicians and clinical pharmacists need to become informed about the usefulness and also the limitations of PGx tests in patient care. Patients and patient advocacy groups also can have significant influence on PGx implementation.

## Challenges for Implementation of PGx

### Providing scientific evidence for improvement in patient care by PGx testing.

On 16 August 2006, a search we did of the medical literature with the MeSH term “pharmacogenetics” on PubMed resulted in 3,347 hits, of which 1,487—almost 45%—were review articles. The relative paucity of original research articles is not the only problem. Many original articles involve a small, specific study population, administration of single doses, use of healthy volunteers instead of patients, or use of a different translation from genotype to phenotype. Moreover, most positive association studies lack validation of findings in an independent patient population.

A classic application of PGx, often used as an example of its potential clinical consequences, involves the variable effect of the antidepressant nortriptyline (NT) due to differences in the gene encoding cytochrome P450 family member CYP2D6. The plasma levels of NT may vary almost 10-fold depending on the number of functional *CYP2D6* alleles. However, the scientific literature reveals a lack of solid evidence that, in the case of NT, the *CYP2D6* polymorphisms actually lead to significant clinical consequences, such as increased toxicity or decreased drug efficacy.

The Pharmacogenetics Working Party of the Royal Dutch Society for the Advancement of Pharmacy is working to implement PGx into their automated medication control database, which is to be used in computerized physician and pharmacist order entry systems (http://farmacogenetica.knmp.nl/). Table 2 summarizes their recently conducted systematic literature search for evidence to define NT dose recommendations for different *CYP2D6* genotype-predicted phenotypes (search terms available upon request).

**Table 2:**
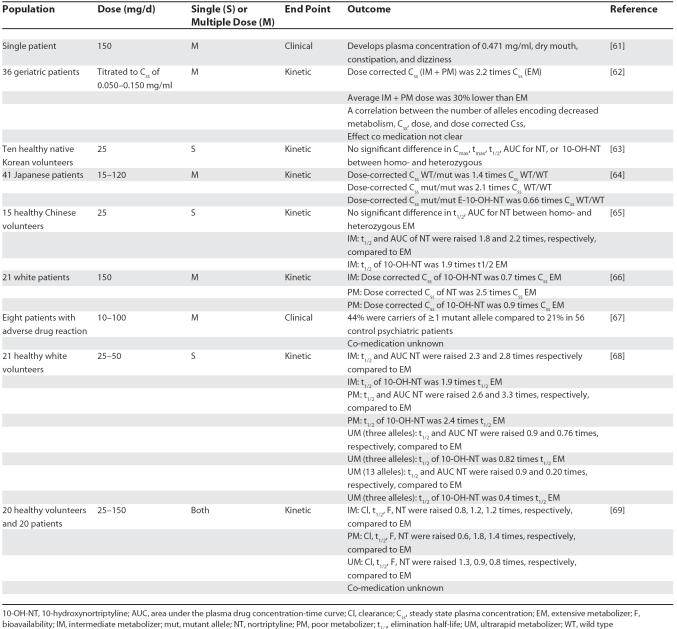
Evidence for Nortriptyline Dose Adjustments Based on PGx

**Box 1. A Matter of Definitions**

In many publications the terms pharmacogenetics (PGt) and pharmacogenomics (PGx) are used interchangeably while others distinguish between the two concepts [54–56]. We prefer to use the single term PGx with the following definition: “the individualization of drug therapy through medication selection or dose adjustment based upon direct (e.g., genotyping) or indirect (e.g., phenotyping) assessment of a person's genetic constitution for drug response.” This definition includes tests operating at protein, metabolite, or other biomarker levels whenever these factors are affected by genetic variation (i.e., single nucleotide polymorphisms, insertions, deletions, microsatellites, variance in copy number, etc). Both germline (i.e., heritable mutations) as well as somatic mutations (i.e., nonheritable mutations in, for example, tumor specimens) are considered. Therefore, immunohistochemical tests such as that for HER2/neu are considered a PGx test in the context of this article.

Only nine scientific articles concerning the interaction between *CYP2D6* and NT, encompassing a total study population of 193 participants, could be retrieved. Among these participants there were only 15 poor metabolizers and 12 ultrarapid metabolizers (UM). Furthermore, the studies frequently were single-dose experiments with healthy volunteers or were limited to specific populations, such as Korean inhabitants or geriatric patients. Most study end points were pharmacokinetic, confirming that *CYP2D6* genotype has an impact on NT pharmacokinetics. However, no drug efficacy or toxicity data were reported. Therefore, even for what is considered a classic example of PGx, solid scientific evidence for clinical relevance is still lacking. In a recent article Kirchheiner et al. [14] provide an overview of how better-designed studies are needed for the clinical breakthrough of PGx and how this breakthrough could be realized by a more systematic inclusion of PGx in drug development.

### Selecting clinically relevant PGx tests.

Research in the field of PGx should be focused on the development of diagnostic tests for clinically important problems. Not every association study leads to a potentially useful PGx test, and financial and technical resources may be wasted if the relevance of more readily measurable values is not excluded first [15]. For example, the 5-hydroxytryptamine 3 receptor antagonists used to prevent nausea and vomiting are known to be metabolized by CYP2D6. Kim et al. showed genotype-dependent pharmacokinetics in healthy volunteers for tropisetron [16], suggesting a hypothesis that cancer patients who are UM are undertreated by a standard dose of tropisetron. This hypothesis was studied by Kaiser and colleagues in 270 cancer patients. Patients with a high number of functional *CYP2D6* alleles experienced more nausea and episodes of vomiting [17]. A similar result was found in patients receiving 4 mg of ondansetron to prevent postoperative nausea and vomiting [18]. These findings clearly show the influence of UM phenotype on both pharmacokinetics and clinical effectiveness of 5-hydroxytryptamine 3 receptor antagonists. However, due to the low prevalence of UM genotype in people of northern European descent, the “number needed to genotype” (i.e., the number of patients needed to genotype in order to prevent one patient from unnecessary nausea and vomiting) appeared to be 50. This number is probably too high to implement this PGx test into routine clinical practice and, more importantly, easier methods such as dose titration or the use of an alternative antiemetic regimen are already available to prevent nausea [19]. PGx studies should be encouraged in fields where the likelihood of a clinically relevant effect is high and its potential usefulness is evident in clinical practice (Table 3).

**Table 3:**
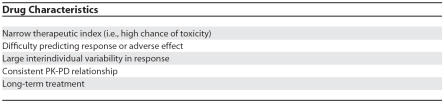
High Likelihood of Clinical Relevance of PGx Test

### Providing data on diagnostic test criteria of PGx testing.

To be clinically useful, a PGx test must predict the outcome of drug treatment. Complex pathways are involved in the action and metabolism of most drugs, and nongenetic influences also contribute to drug response [15]. Therefore, PGx testing for single polymorphisms may account for only part of the variability in drug response. The diagnostic test criteria sensitivity, specificity, and predictive value are applicable to tests for which response is determined as a dichotomous variable. However, drug response cannot always be considered an all-or-none phenomenon. In these situations the relative contribution of the genotype to the variability in response (the percentage explained variance, *R* ^2^) provides additional information. Diagnostic test criteria of PGx tests are not commonly reported, but are important for clinical implementation. Table 4 summarizes the characteristics of selected PGx tests.

**Table 4:**
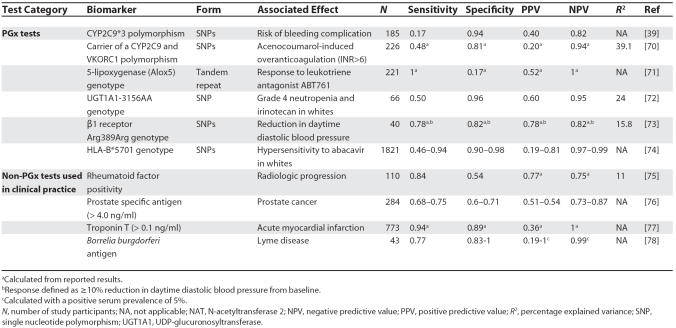
Comparison of Diagnostic Test Criteria of a Selection of PGx Tests and Non-PGx Tests Used in Clinical Practice

**Box 2. PGx Need Not Be Financially Unattractive from a Drug Manufacturer's Point of View**

The potentially smaller market for a drug could be compensated by (1) an increased rate of adoption of the drug; (2) the identification of patients who otherwise would not have been candidates for the drug; (3) increased compliance with improved efficacy; and (4) the possibility of premium pricing [57]. This process can be illustrated with preliminary calculations of the use of the tumor necrosis factor alpha-blocking drug adalimumab used in the treatment of rheumatoid arthritis.

The prevalence of rheumatoid arthritis in adults in The Netherlands is 1%, resulting in approximately 160,000 potential users of adalimumab. The estimated cost for the treatment of all these patients with adalimumab during one year is about €1,900,000,000. To limit the costs, the use of adalimumab has been restricted to treatment of patients with moderate to severe rheumatoid arthritis failing to respond on disease-modifying antirheumatic drugs or methotrexate. As a result, only 3,440 patients, or 2.15% of the potential 160,000, used the drug in 2005. When a certain PGx test enables predicting the response to adalimumab, there would be no legitimate reason to withhold the drug from the predicted responders; and if the prevalence of the responsive genotype were to exceed 2.15% in the rheumatoid arthritis patient population the revenues of the manufacturer would increase.

It can be observed that the diagnostic test criteria for PGx tests are comparable to those of clinically available non-PGx tests (also shown in Table 4). Thus, while some consider current PGx tests as having inadequate value for clinical application, tests with comparable diagnostic test criteria are currently being used in patient care. The need for well-defined PGx test criteria has been previously discussed [20,21]. We maintain that demonstration of potential clinical usefulness requires the reporting of diagnostic test criteria in PGx association studies.

### Providing information on cost-effectiveness and cost-consequences of PGx testing.

Although funding agencies including health insurers have funded many PGx research projects in recent years, their willingness to reimburse routine PGx testing will require information on cost-effectiveness and cost-consequences. In 2004, Phillips performed a systematic literature review on cost-effectiveness of PGx testing [22]. Only 11 published true cost-effectiveness analyses (CEAs) could be retrieved. Seven studies found a PGx-based strategy to be cost-effective, two showed equivocal results, and two concluded that a PGx-based strategy was not cost-effective. Despite the publication of additional CEAs of PGx, there is a need for more information [23–26]. The performance of such CEAs is problematic for two reasons. First, there are limited data on the rate at which PGx testing actually prevents adverse drug reactions. Second, PGx test prices are dropping continuously. Even without data from a comprehensive CEA, some simple calculations can be made and preliminary conclusions can be drawn on potential cost-effectiveness of PGx testing (Box 3).

The example in Box 3 indicates that screening for dihydropyrimidine dehydrogenase (DPD) deficiency in all 5-fluorouracil (5FU)-treated patients is not cost-effective, mainly due to the low incidence of DPD deficiency and the high cost of the phenotypic assay. It might become cost-effective if the cost of the assay decreases. Circumstances that favor the cost-effectiveness of PGx testing include high prevalence of the relevant allelic variant in the target population, good correlation between genotype and phenotype, satisfactory diagnostic test criteria, phenotype associated with significant morbidity or mortality if left untreated, and significant reduction in adverse drug reactions reduction by PGx testing [27].

Although the necessity of CEAs for every new clinical technique is debatable, and several innovations have found their way to application without proof of their cost-effectiveness [28,29], more research on the cost-effectiveness and cost-consequences of PGx testing will nonetheless stimulate its further implementation into clinical practice.

### Developing guidelines directing the clinical use of PGx test results.

PGx studies published to date usually report that carriers of a specified genotype in a particular patient population have an increased likelihood of a desired (or undesired) outcome of drug treatment. Such studies have not, however, resulted in the distillation of practical prescribing recommendations based on genotype. In particular, very little data are available on effective and safe dose adjustment for the different metabolizer phenotypes, although a 2001 consensus paper on deriving CYP2D6 phenotype-related dose recommendations for antidepressants from pharmacokinetic study data represents an early step [30]. Coumarins used in the treatment and prevention of venous and thromboembolic disorders constitute one case in which the application of dose recommendations is relatively far advanced. Coumarins (e.g., warfarin, phenprocoumon, acenocoumarol) are primarily metabolized by CYP2C9, and treatment outcome is known to be associated with *CYP2C9* genotype [31–39]. More recently, the gene coding for the vitamin K epoxide reductase subunit 1 (VKORC1) was found to contribute to the variability in response observed in warfarin users [40].

The effect of *CYP2C9* and *VKORC1* genotype combined with patient height explained up to 55% of variance in warfarin dose [41]. Two prospective (pilot) studies concluded that the use of an algorithm including *CYP2C9* genotype for warfarin dosing is feasible [42,43], and prospective research is ongoing in the UK. Therefore, prospectively validated coumarin dosing algorithms that include PGx information might become available in the near future. In more recent developments, Wessels et al. have developed a clinical scoring system based on seven factors, including four genetic polymorphisms, to predict efficacy of methotrexate monotherapy in rheumatoid arthritis patients. They provide a tool that translates the outcome of the model into individual treatment recommendations [44]. De Leon et al. have published clinical guidelines for using *CYP2D6* and *CYP2C19* genotypes in the prescription of antidepressants or antipsychotics [45]. Further translational research aimed specifically at the practical application of PGx in clinical situations is warranted.

### Improving acceptance of PGx testing.

A newly introduced drug or technology is normally first applied by a small group of clinicians. In time it may become standard treatment incorporated into guidelines and consequently into wider clinical use. The time from introduction to acceptance of new methods may vary widely, as illustrated by a comparison of the implementation of Calvert's formula with that of HER2/neu testing. Carboplatin is currently dosed using the formula of Calvert, published in 1989, for area-under-the-curve targeted dosing [46]. Attention was called to Calvert's formula several times but it was not until 1996 that it was reported by the American Hospital Formulary Service, a widely used source of drug information [47,48]. Assuming that uptake into guidelines to some extent represents clinical acceptance, this time course shows that it took no less than seven years for Calvert's formula to be accepted. This relatively slow acceptance is further exemplified by the limited use of the formula in clinical trials with carboplatin during the early 1990s (Figure 2).

**Figure 2:**
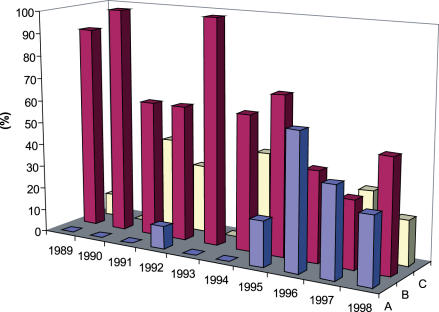
The Use of the Calvert Formula in Clinical Trials from 1989 to 1998 A PubMed search for the dosing of carboplatin in clinical trials was performed for the period 1989–1998. For each year the first ten results of PubMed were screened for the use of the Calvert formula. Bars represent the percentage of results in which the Calvert formula was used to dose carboplatin (A), the Calvert formula was not used (B), or no dosing information could be retrieved electronically (C).

**Box 3. Estimated Potential Cost-Effectiveness of DPD Screening**

The cytotoxic drug 5FU is widely used, for example in colorectal cancer. Severe neutropenia is associated with deficiency of the enzyme DPD, which metabolizes 5FU [58]. The deficiency of DPD is thought to be caused by germline mutations in the gene encoding DPD.

A possible strategy would be to test all 5FU-treated patients, and we estimate the cost consequences for the Dutch situation as follows. About 7,000 patients per year are treated with 5FU. A phenotypic test measuring DPD activity in peripheral mononuclear cells is available, and normal values for enzyme activity in both wild-type and heterozygotes are known, but are relatively difficult to distinguish. The incidence of DPD deficiency is about 3% and, therefore, 210 patients of the 7,000 5FU-treated patients may be detected by this test [59].

In a meta-analysis on 5FU-related toxicity it was reported that the incidence of 5FU-related death is about 0.5%, and in 50% of the cases toxicity was explained by deficiency of the enzyme DPD [60]. The cost of the DPD assay is €850, which would result in an estimated cost of nearly €6 million to test all 7,000 patients for DPD status. This testing would save 17 patients per year, at a cost of €350,000 per saved life, which may be unrealistically high. Moreover, even then, 17 other patients will die from 5FU-related toxicity anyway, because their toxicity is not related to DPD deficiency. Although this example is evaluated in a Dutch setting the data and conclusion can be applied to other settings.

A contrasting example is the implementation of testing of breast cancers for HER2/neu overexpression with immunohistochemistry or fluorescence in situ hybridization to select patients with metastasized breast cancer eligible for treatment with trastuzumab. In the late 1980s and early 1990s, several studies demonstrated that breast cancers with HER2/neu overexpression showed poor prognosis [49–53]. In 1998 trastuzumab, a monoclonal drug directed against the HER2 protein, was launched on the US market. One year later, testing for HER2/neu overexpression was included in the American Hospital Formulary Service trastuzumab monograph. Testing for HER2/neu overexpression has become standard practice for guiding drug therapy for metastatic breast cancer. In contrast to the lengthy time line for acceptance of Calvert's formula, the short time line of acceptance of testing for HER2/neu overexpression indicates that fast uptake is possible. The two examples differ in many respects (e.g., one results in a dose adjustment while the other results in the decision whether or not to prescribe the drug). Nonetheless, two differences might be observed to present potential opportunities for improved clinical uptake of PGx. First, the use of testing for HER2/neu overexpression was required by the regulatory agencies upon market introduction of trastuzumab. With regard to PGx testing, this requirement suggests that obligatory testing prior to drug prescribing might give a strong stimulus to the clinical uptake of PGx. Second, HER2/neu testing was actively advocated by the pharmaceutical company manufacturing the drug and by patient advocacy organizations. Similarly active support for the use of clinically established PGx tests by pharmaceutical companies or patient advocacy organizations might be expected to improve clinical uptake of PGx testing.

## Conclusions

Because variation in drug responses is, at least to some extent, related to genetic variation, PGx testing has the potential to result in safer and more effective use of drugs by permitting individualized therapy. In recent years FDA-approved PGx tests have become available, but the use of PGx testing has remained limited, largely by a lack of scientific evidence for improved patient care by PGx testing. Providing this scientific evidence presents a significant challenge. The development of novel tests should be aimed at solving important clinical problems. To demonstrate potential for clinical use, PGx studies should report diagnostic test criteria. For PGx tests shown to improve patient care, guidelines directing the clinical use of PGx test results should be developed. Information on cost-effectiveness and cost-consequences of PGx testing should be provided to facilitate reimbursement by insurance companies. Finally, uptake in clinical practice will be given a stimulus if regulatory agencies recommend testing prior to prescribing the drug, and if pharmaceutical companies or patient groups advocate for use of the test. If the outlined challenges can be met, the incorporation of PGx in routine clinical practice may prove an achievable goal in the near future.

## Abbreviations

5FU: 5-fluoruracil
CEA: cost-effectiveness analysis
DPD: dihydropyrimidine dehydrogenase
FDA: US Food and Drug Administration
NT: nortriptyline
PGx: pharmacogenomics/pharmacogenetics
UM: ultrarapid metabolizer(s)
